# Hyperthermia enhances photodynamic therapy by regulation of HCP1 and ABCG2 expressions via high level ROS generation

**DOI:** 10.1038/s41598-018-38460-z

**Published:** 2019-02-07

**Authors:** Hiromi Kurokawa, Hiromu Ito, Masahiko Terasaki, Hirofumi Matsui

**Affiliations:** 1 Faculty of Medicine, University of Tsukuba, 305-8575 Ibaraki, Japan; 2 Graduate School of Medical and Dental Sciences, Kagoshima University, 890-8544 Kagoshima, Japan; 3 Graduate School of Comprehensive Human Sciences, University of Tsukuba, 1-1-1 Ten-nohdai, Tsukuba, Ibaraki 305-8575 Japan

## Abstract

Photodynamic therapy (PDT) is a cancer treatment that make use of the cancer-specific accumulation of porphyrins. We have reported that mitochondrial reactive oxygen species (mitROS) upregulate uptake transporter of porphyrins, heme carrier protein-1 (HCP-1). The accumulation of cancer-specific porphyrins was increased by mitROS production, thereby the cancer-specific PDT cytotoxicity was enhanced. Thus we investigated whether mitROS production by hyperthermia can enhanced the cytotoxicity of PDT or not. In this study, 1 h of hyperthermia at 42 °C increased the mitROS production, and both the accumulation of cancer-specific porphyrins and the PDT cytotoxicity increased. Moreover, the authors treated cells with N-acetyl-L-cysteine (NAC) to examine the effect of mitROS. NAC inhibited the increasing ROS production after hyperthermia to restrain the post-treatment increase of cancer-specific porphyrins accumulation. Moreover, the increase of ROS production in cancer cells after hyperthermia upregulated HCP-1 expression and downregulated ABCG2 expression. These regulation were inhibited by NAC. These results suggest that hyperthermia treatment increased mitROS production, which involved HpD accumulation and enhanced PDT effects in cancer cells. The mechanism of this phenomenon was most likely to be due to both the upregulation of HCP-1 and the downregulation of ABCG2 by mitROS.

## Introduction

The effects of photodynamic therapy (PDT) are strongly influenced by the accumulation of cancer-specific porphyrins. We have previously focused on the mechanism for cancer-specific accumulation of porphyrins, and demonstrated that heme carrier protein-1 (HCP-1), a heme transporter^1,2^, was overexpressed in cancer cells compared to normal cells, resulting in increased transport of porphyrins into the cells^3^. Furthermore, HCP-1-overexpressing HeLa cells had enhanced hematoporphyrin dihydrochloride (HpD) accumulation and phototoxicity of PDT, whereas HpD accumulation in HCP-1 knockdown cells were decreased^3^. It is well known that levels of reactive oxygen species (ROS) are higher in cancer cells compared to normal cells because of mitochondrial dysfunction^4,5^. We also reported that mitochondrial ROS (mitROS) were one of the factors that enhanced tumor invasion in gastric cancer cells while also regulating HCP-1 expression^6,7^. In our previous study, we used the three following cell lines: a rat gastric mucosa cells (RGM1), the cancerous version of RGM1 cells (RGK1), and manganese superoxide dismutase-overexpressing cells (RGK-MnSOD)^7–9^. As MnSOD is a mitochondrial antioxidant enzyme that converts superoxide into oxygen or hydrogen peroxide^10^, mitROS in RGK-MnSOD should be scavenged. Using these cell lines, we demonstrated that HCP-1 expression in RGK1 cells was higher than that in RGK-MnSOD or RGM1 cells. Additionally, PDT cytotoxicity in RGK1 cells was also higher^6^. Thus, we proposed that increasing mitROS most likely enhances the PDT effect.

Hyperthermia is also a non-invasive cancer therapy that is similar to PDT. During the treatment, the tissue temperature should be maintained between 41–44 °C. This temperature range does not cause cytotoxic damage to normal cells, while does show cytotoxicity to cancer cells; this difference has been reported to be due to the underdeveloped vascular system specific to cancer cells^11^. There are three methods for hyperthermia: local, regional, and whole-body hyperthermia^12^. In local hyperthermia, the tissue temperature is kept between 41–42 °C in a small area using microwaves, radiofrequency, and ultrasound. In regional hyperthermia, the body cavity, organ, or limb are heated. In whole-body hyperthermia, the body temperature is raised to 42 °C using an aquatherm or iratherm system. Compared to 37 °C, 42 °C produces a mild heat stress for the cells and thus superoxide anions are released from the tissue^13^. Superoxide anions have been reported to be produced by the mitochondrial electron transport chain^14^.

Depending on the type of oncogenic mutations, the phenotypic heterogeneity of cancer cells can show various responses to drug treatments^15^. Indeed, clones derived from the mouse breast cancer cell line 4T1 showed diverse drug response patterns and heterogeneous phenotypes^16^. We also estimated several RGK1 sub-clones using the limited dilution method. Clones had different characteristics such as ROS or NO generation and tumorigenesis. Cancer stem cells showed resistance to conventional anti-cancer therapies and increased metastases or tumor recurrence^17^. Furthermore, cancer stem cells were also involved in the reconstitution of the tumor microenvironment through trans-differentiation into different lineages^18^. Overall, cancer heterogeneity may be due to the plasticity of cancer stem cells^19^.

In this study, we investigated the effects of combination therapy with both hyperthermia and PDT. We also investigated the mechanism of this combination therapy using RGK1 sub clones, which show different characteristics.

## Results

### The characteristics of RGK36 and RGK45 cells

**Figure 1:**
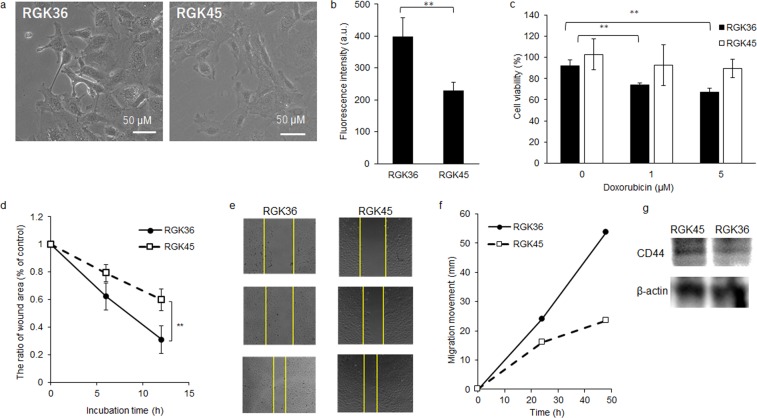
The characteristics of RGK36 and RGK45 cells. (**a**) Phase contrast imaging. (**b**) Fluorescence intensity of DAF2-DA. (**c**) Drug resistance for doxorubicin. (**d**) Representative pictures showing the wound at 0, 6, and 12 h. (**e**) The percentage of wound healing. (**f**) Invasion assay using Matrigel. (**g**) CD44 expression. Data are expressed as means ± SD (n = 4). *p < 0.05, **p < 0.01.

**Figure 2:**
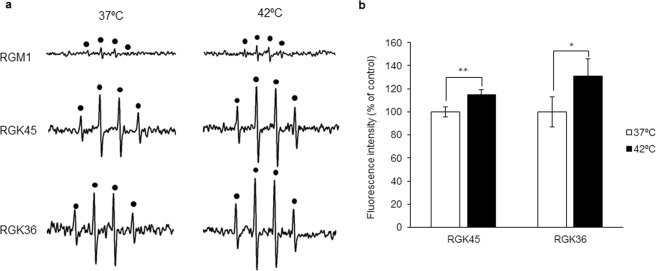
Intracellular ROS production after treatment with or without hyperthermia treatment. (**a**) The ESR spectra from RGK36 and RGK45 cells were increased after the hyperthermia treatment, while RGM1 cells were not. (**b**) MitoSOX intensity in RGK 36 and RGK45 cells were elevated after the hyperthermia treatment. MitoSOX was excited at 510 nm, and the emission was collected at 580 nm. Data are expressed as means ± SD (n = 4). *p < 0.05, **p < 0.01.

The characteristics of RGK36 and RGK45 cells (Fig. 1a) were demonstrated by the six following experiments: DAF-2DA, electron spin resonance (ESR), drug resistance, wound healing assay, cellular invasion assay, and CD44 expression. Intracellular NO and ROS were evaluated by DAF-2DA staining and ESR, respectively. NO and ROS production in RGK36 cells were higher than that in RGK45 cells (Figs 1b and 2a). The drug resistance for doxorubicin in RGK36 and RGK45 cells was examined with the MTT assay. Cells were incubated with 1 or 5 μM doxorubicin for 24 h. The cell viability of RGK36 cells significantly decreased after doxorubicin treatment, while that of RGK45 cells showed no significant effect (Fig. 1c). The horizontal cellular migration was evaluated by the wound healing assay in which the results were influenced by the cell growth. After 12 h, RGK36 cells showed a better recovery than RGK45 cells (Fig. 1d,e). We measured the cellular invasive depth of both RGK36 and RGK45 cells from the Matrigel surface. After 48 h, the depth of the RGK36 cells was 324 µm, whereas that of RGK45 cells was 213 µm. The invasive ability of RGK36 cells was significantly higher than that of RGK45 cells (Fig. 1f). CD44 is a cancer stem cell marker, and its expression in RGK45 cells was higher than that in RGK36 cells (Fig. 1g).

### Hyperthermia induced mitochondrial ROS generation in cancer cells

The generation of ROS in cells with or without hyperthermia treatment was evaluated using ESR. The ESR spectral intensity in RGK36 and RGK45 cells were increased after the hyperthermia treatment. Compared to RGM1 cells without the hyperthermia treatment, the ESR spectral intensity of cells with the hyperthermia treatment was almost the same (Fig. 2a). Using MitoSOX, which is a mitochondrial superoxide indicator, mitROS were also elevated by hyperthermia treatment in RGK36 and RGK45 cells (Fig. 2b).

### Hyperthermia enhanced intracellular HpD accumulation and cytotoxicity of PDT in cancer cells

**Figure 3:**
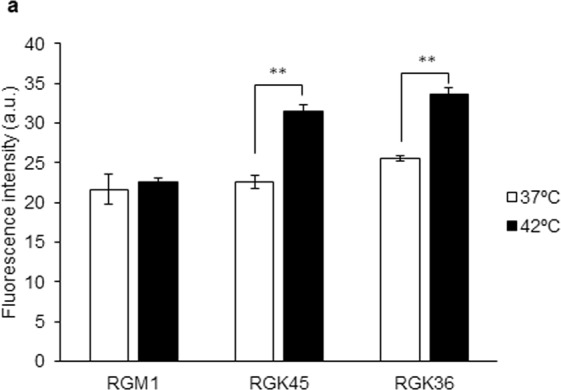
Intracellular HpD fluorescence intensity after treatment with or without hyperthermia treatment. Cells were exposed to culture medium containing 20 μM of HpD for 6 h, and the fluorescence of HpD incorporated into the cells was measured on a microplate reader. HpD was excited at 415 nm, and the emission was collected at 625 nm. Data are expressed as means ± SD (n = 4). *p < 0.05, **p < 0.01.

**Figure 4:**
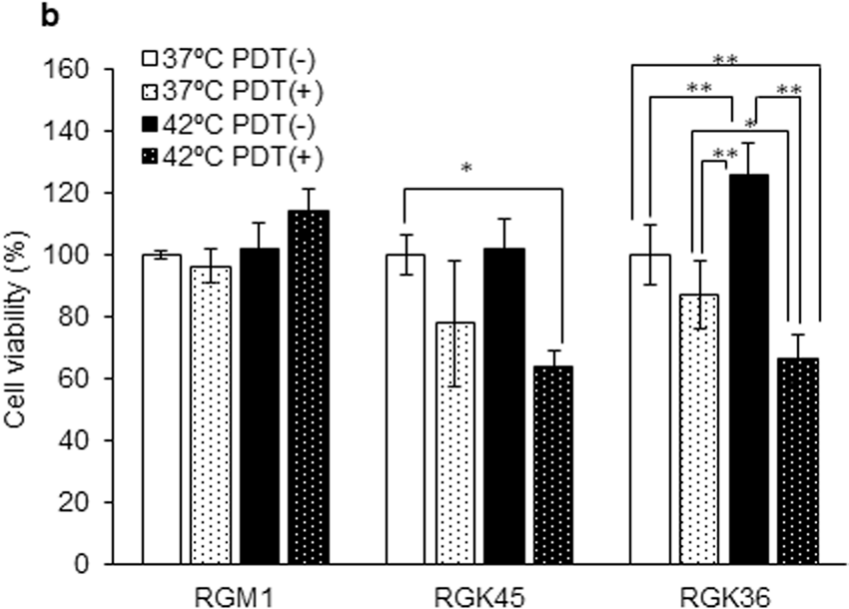
The effect of combination therapy with both hyperthermia and PDT. After treatments with or without hyperthermia, cells were incubated with 20 μM HpD for 24 h. Next, cells were irradiated with the excimer dye laser light. HpD was excited at 630 nm. Data are expressed as means ± SD (n = 4). *p < 0.05, **p < 0.01.

HpD accumulation increased due to the hyperthermia treatment in both RGK36 and RGK45 cells. However, HpD accumulation in RGM1 cells did not change with hyperthermia treatment compared to those without the treatment (Fig. 3). The enhanced effects of PDT by hyperthermia was measured using the WST assay, which is an alternative method to the MTT assay. RGK36 and RGK45 cells were injured by PDT, while RGM1 cells were not. Moreover, the PDT effect for RGK36 and RGK45 cells were enhanced by 24 h after the hyperthermia treatment (Fig. 4).

### NAC decreased intracellular ROS production and HpD accumulation by hyperthermia

**Figure 5:**
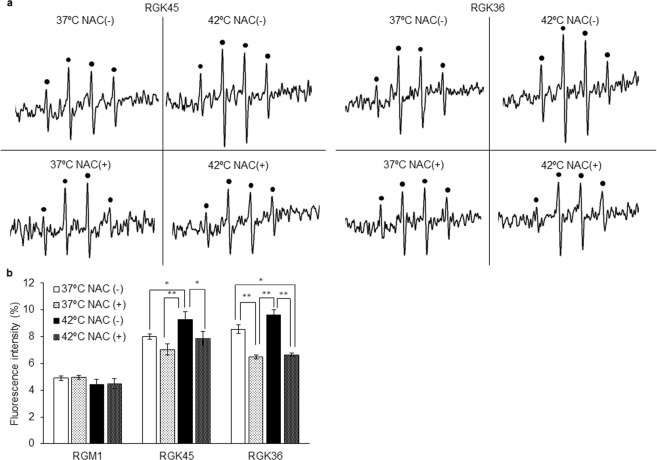
The effects of NAC on intracellular ROS production and HpD accumulation. (**a**) The ESR signal indicated intracellular ROS production. (**b**) After hyperthermia treatment with or without NAC, cells were exposed to culture medium containing 20 μM of HpD for 6 h. Intracellular fluorescence intensity was measured on a microplate reader. Data are expressed as means ± SD (n = 4). *p < 0.05, **p < 0.01.

ROS generation in RGK36 and RGK45 cells was measured using ESR. Intracellular ROS generation in both cell lines increased by hyperthermia treatment and decreased by N-acetyl-L-cysteine (NAC) treatment. Furthermore, ROS production in cells due to hyperthermia treatment with NAC was inhibited and was comparable to cells without the hyperthermia treatment (Fig. 5a). Moreover, HpD accumulation was also inhibited by NAC in cancer cells (Fig. 5b). HpD accumulation by hyperthermia treatment had no effect on normal cells.

### Hyperthermia regulated the expression of transporters and NAC inhibited the effects of hyperthermia

**Figure 6:**
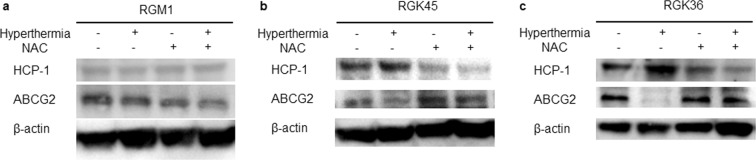
Western blot analysis of HCP-1 and ABCG2 expressions in (**a**) RGM1, (**b**) RGK45, and (**c**) RGK36 cells. The HCP-1 and ABCG2 expressions in RGK45 and RGK36 cells were affected by the hyperthermia or NAC treatment when compared with RGM1 cells.

The expressions of transporters in cells with or without the hyperthermia treatment were analyzed by western blotting. In RGK36 and RGK45 cells, HCP-1 expressions were increased, while the expressions of ATP-binding cassette sub-family G member 2 (ABCG2), which exports porphyrins and some anti-cancer drugs from cells, were decreased by the hyperthermia treatment. Moreover, NAC, a radical scavenging accelerator, decreased the expression of HCP-1 and increased the expression of ABCG2. In contrast, HCP-1 and ABCG2 expressions were the same in RGM1 cells with or without hyperthermia or NAC treatments (Fig. 6).

## Discussion

In this study, we demonstrated for the first time that a hyperthermia pretreatment increased intracellular HpD accumulation, thereby enhancing cancer-specific PDT cytotoxicity by the upregulation of ROS production. We also demonstrated that the increased ROS production by hyperthermia involved either the upregulation of HCP-1 expression or the downregulation of ABCG2 expression.

It is well known that hyperthermia treatment increases intracellular ROS production^20,21^. In this study, 1 h of hyperthermia at 42 °C increased the ESR signal intensity in cancer cells (Fig. 2a). Analyses with MitoSOX demonstrated that the increased ROS levels were derived from the mitochondria (Fig. 2b). MnSOD is an enzyme that scavenges mitochondrial ROS^7^. In our study, MnSOD overexpression in cancer cells decreased HpD accumulation, thereby attenuating the effects of PDT^6^. From these results, we concluded that the hyperthermia treatment increased mitROS production, which involved HpD accumulation and enhanced PDT effects in cancer cells. We also investigated the effects of NAC, a ROS scavenger, on HpD accumulation^22^, which resulted in decreased ROS generation in RGK36 and RGK45 cells. NAC also attenuated the increase in ROS production from the hyperthermia treatment (Fig. 5a). Moreover, NAC inhibited the accumulation of cellular porphyrins (Fig. 5b). From these results, we proposed that the control of mitROS levels can regulate cancer-specific porphyrin accumulation.

The synergic effect of both hyperthermia and PDT have been previously investigated by Yanase *et al*. To confirm this combination treatment, the authors used an irradiator that had two filters, allowing for the generation of two types of rays. The first type generated waves of 580–740 nm to excite protoporphyrin IX, while the second type generated waves of 940–1,600 nm to increase temperature^23^. Corato *et al*. synthesized liposomes containing both magnetic nanoparticles and a photosensitizer, which was then used to demonstrate that the effects of a combination therapy with both hyperthermia and PDT was synergistic *in vitro* and *in vivo*^24^. He *et al*. reported that the upregulation of HSP70 expression activated cytotoxicity of CD8 T cells. In those conditions, the effects of PDT was enhanced^25^. In this study, we also confirmed that the effects of PDT in cancer cells were enhanced by hyperthermia. We have previously confirmed that HCP-1 was not only a heme transporter, but also a porphyrin transporter, which was upregulated by mitROS^6^. As hyperthermia enhanced mitROS production in cancer cells (Fig. 2), HCP-1 expression was increased. Furthermore, we also confirmed the expression of HCP-1 in RGK36 and RGK45 cells, which are gastric cancerous mutant cells. Moreover, NAC decreased HCP-1 expressions and ROS productions, and attenuated the increase in HCP-1 due to hyperthermia (Fig. 6b,c). We proposed that the level of HCP-1 expression was dependent on ROS levels.

To maintain the concentration of a substrate, accumulating transporters and excreting transporters are necessary. Porphyrins in cancer cells are important for effective and specific treatments. ABCG2 (BCRP) excretes porphyrins to maintain intracellular porphyrin homeostasis^26,27^. In this study, we investigated whether ROS can regulate ABCG2 expression levels. Contrary to HCP-1, ABCG2 expression was downregulated by hyperthermia treatment via increased ROS production (Fig. 6b,c). Similar to the effects of NAC, the expression of ABCG2 was upregulated by decreasing ROS production. A relationship between ABCG2 and ROS has also been previously reported: Scoparo *et al*. reported that hispidulin increased ROS production and inhibited the ABCG2-mediated efflux of mitoxantrone^28^. Mason *et al*. also reported that prostaglandin E2, which has a positive correlation with ROS and PGE2^20,29,30^, downregulated ABCG2 expression^31^. From these results, we proposed that mitROS involved both the upregulation of HCP-1 and downregulation of ABCG2.

We have previously established the rat gastric cancer mutant cell line RGK-1 induced by N-methyl-N′-nitro-N-nitrosoguanidine^9^, and thereafter performed cloning by limiting dilution for polyclonal RGK-1 cells. RGK36 and RGK45 cells are RGK1-derived sub-clone cells, and then we compared the characteristics of the RGK36 and RGK45 cells. RGK36 cells showed higher intracellular ROS production and cell proliferation, whereas RGK45 cells showed higher drug resistance and CD44 expression (Fig. 1). Thus, we determined RGK36 cells to be highly mature cancer cells, while RGK45 cells to be cancer stem cell-like cells. Cancer stem cells are believed to be the cause of chemo- and radio-resistance, which involves metastasis and recurrence in many types of cancer^32^. Hence, many researchers have studied the mechanism for these phenomena to create complete therapies for cancer stem cells^33,34^. In this study, hyperthermia enhanced the effects of PDT for RGK45 cells. Hyperthermia treatment downregulated ABCG2 expression in RGK45 as a result of an increased in ROS levels in cancer stem cells. Accordingly, we proposed that hyperthermia treatment can be used to address drug resistance in cancer stem cells.

In conclusion, hyperthermia treatment enhanced the cancer-specific effects of PDT. We proposed that the mechanism of this phenomenon was most likely to be due to the upregulation of HCP-1 and downregulation of ABCG2 by mitROS.

## Methods

### Cell culture and hyperthermia treatment

A rat gastric epithelial cell line RGM-1 and its chemically oncogenic cancer-like mutant cell lines (RGK36 and RGK45) were cultured in DMEM/F12 with L-glutamine (Life Technologies Japan Ltd., Tokyo, Japan), and DMEM/F12 without L-glutamine (Sigma-Aldrich Japan K.K., Tokyo, Japan), respectively. The culture media contained 10% inactivated fatal bovine serum (Biowest LLC, Kansas City, MO) and 1% penicillin/streptomycin (Wako Pure Chem. Ind. Ltd., Osaka, Japan). All cells were cultured in 5% CO_2_ and 37 °C. Cells were seeded onto dishes or plates under culture conditions overnight before treatment. Cells were incubated at 37 °C or 42 °C with or without 10 mM NAC for 1 h. For recovery after treatments, the cells were cultured in 5% CO_2_ at 37 °C until further analysis.

### NO detection with DAF-2DA

RGK36 and RGK45 cells were cultured on 96-well plates at a density of 5 × 10^3^ cells/well and incubated overnight. After incubation, the supernatant was aspirated, and the cells were incubated then with 10 μM DAF-2DA (Daiichi Pure Chemicals, Tokyo, Japan) for 30 min. Cells were then rinsed twice with Hanks balanced salt solution (HBSS), and the fluorescence intensity of DAF-2DA was measured by a Varioskan microplate reader. Excitation and emission wavelengths were 495 and 515 nm, respectively.

### Wound healing assay

RGK36 and RGK45 cells were seeded onto 12-well plates at a density of 1 × 10^5^ cells/well. Cells were incubated until 85% confluency and then wounded using a yellow pipette chip. After incubating for 0, 6, and 12 h, images of the wound closure were obtained on a BZ-X700 microscope (Keyence, Osaka, Japan) and analyzed using the imageJ software.

### Cell viability assay

Cell viability assay was demonstrated using the Cell Counting Kit-8 (Dojindo, Tokyo, Japan) according to the manufacturer’s protocol. Cells were cultured on 96-well plates at a density of 5 × 10^3^ cells/well and incubated overnight. The supernatant was then aspirated and medium containing 1 or 5 μM doxorubicin (Wako Pure Chem.) was added. Cells were then incubated at 37 °C for 24 h. Next, cells were incubated with 10% Cell Counting Kit-8. The absorbance at 450 nm was measured by a DTX880 multi-mode microplate reader (Beckman Coulter, Inc., Brea, CA).

### Tumor invasion assay

The tumor invasion assay was performed according to a previous report^7^. A Matrigel (Corning Inc., NY, USA) matrix was created in the wells of a 96-well plate. Matrigel solutions were poured to the well on ice, and then the plate was incubated at 37 °C for 30 min. After polymerization of the Matrigel, RGK36 and RGK45 cells were seeded at a density of 500 cell/well and incubated for 24 h. The invasion length was measured on a BZ-X700 microscope.

### ESR spectroscopy

ROS generation in cells was measured by ESR according to a previous study^35^. RGK36, RGK45, and RGM1 cells were cultured on a glass cover slide (49 × 5 × 0.2 mm) until the cells were confluent. Cells were incubated at 37 or 42 °C for 1 h and then immersed in respiratory buffer containing 5 mM succinate (Sigma-Aldrich Japan K.K.), 5 mM malate (Wako Pure Chem. Ind.), 5 mM glutamate (Sigma-Aldrich Japan K.K.), 5 mM nicotinamide adenine dinucleotide (Sigma-Aldrich Japan K.K.), and 10 mM 5,5-dimethyl-1-pyrroline-N-oxide (DOJINDO). The cell-attached glass cover slide was placed on a tissue glass, and the ESR spectra were obtained by inserting the tissue glass into the device. All ESR spectra were obtained using a JEOL-TE Xband spectrometer (JEOL Ltd., Tokyo, Japan) under the following conditions: 7.5 mT sweep width, 1000 gain, 0.1 mT modulation width, and 10 mW incident microwave power.

### Measurement of mitochondrial ROS

Mitochondrial ROS was detected using the fluorescence indicator MitoSOX (Life Technologies Inc.). RGK36 and RGK45 cells were incubated at 37 or 42 °C for 1 h and then incubated for 30 min in 5 μM MitoSOX diluted with HBSS. After incubation, the cells were washed three times with HBSS. Fluorescence intensity of MitoSOX was measured by a Varioskan micro plate reader (Thermo Fisher Scientific K.K., Kanagawa, Japan). The measurement wavelengths of excitation and emission were 510 and 580 nm, respectively.

### Cellular uptake of HpD

RGK36, RGK45, and RGM1 cells were incubated overnight in 12-well plates at 5 × 10^4^ cells/well. Cells were incubated at 37 or 42 °C for 1 h and then incubated at 37 °C for 24 h. After treatments, cells were incubated with 20 μM HpD for 6 h and then rinsed with PBS and lysed in 100 μL self-prepared RIPA buffer^36^. Cell homogenates were then transferred to a 96-well plate. The fluorescence intensity of HpD was measured by a Varioskan microplate reader (Thermo Fisher Scientific K.K.). Excitation and emission wavelengths were 415 and 625 nm, respectively.

### Western blot analyses

RGK36, RGK45, and RGM1 cells were incubated overnight in 60-mm dishes. Cells were incubated at 37 or 42 °C for 1 h and then incubated at 37 °C for 24 h. Whole cell lysates were prepared by rinsing the cells three times with PBS, adding RIPA buffer containing protease inhibitor cocktail (Thermo Fisher Scientific) on ice, then boiling at 95 °C for 10 min. For SDS-polyacrylamide gel electrophoresis, the cell lysates were added into wells of NuPAGE® Novex® 4–12% Bis-Tris gels (Thermo Fisher Scientific K.K.). The gels were electrophoresed at 100 V for 70 min, and proteins were transferred onto a PVDF membrane (Bio-Rad Laboratories, CA, USA) by electrophoresis at 1.2 mA/cm^2^ for 60 min. The membrane was blocked for 60 min with PVDF blocking reagent from the Can Get Signal® (TOYOBO CO. LTD., Osaka, Japan) and probed with primary and secondary antibodies. Anti-rabbit HCP-1 (Abcam plc., Cambridge, U.K.) or ABCG2 (Cell Signaling Technology Japan K.K., Tokyo, Japan) antibodies (1:1000) were added to the Can Get Signal® Immunoreaction Enhancer Solution 1 (TOYOBO), and then exposed to the membrane overnight. After the primary antibody solution was aspirated, the membrane was washed three times with 1 × tris-buffered saline containing Tween 20. The secondary HRP-linked anti-rabbit IgG antibody (Cell Signaling Technology Japan K.K.) (1:1000) was added to the Can Get Signal® Immunoreaction Enhancer Solution 2 (TOYOBO) and exposed to the membrane for 60 min. Lumina forte western HRP substrate (Millipore Co., Billerica, MA) was used to visualize the membrane. Images of the blots were captured on the ImageQuant LAS4000 (GE Health Care Japan, Tokyo, Japan). β-Actin (Cell Signaling Technology Japan K.K.) was detected as the control for protein loading.

### Cell viability assay after PDT

RGK36, RGK45, and RGM1 cells were incubated overnight in 96-well plates at 1 × 10^3^ cells/well. Cells were incubated at 42 °C for 1 h and then incubated at 37 °C for 24 h. After treatments, cells were incubated with 20 μM HpD for 24 h and then rinsed with PBS. The medium was exchanged with fresh medium lacking phenol red. Cells were irradiated with an excimer dye laser light (630 nm, 0.5 J/cm^2^) for PDT via PDT EDL-1 (Hamamatsu Photonics K.K., Hamamatsu, Japan). After the irradiation, cells were incubated for 24 h. The medium was replaced with fresh medium containing 10% of Cell Counting Kit-8 and then further incubated. The absorbance at 450 nm was measured on a DTX880 multi-mode microplate reader.

### Statistical analysis

Data are expressed as means ± SD and were assessed by analysis of variance. Individual groups were compared by Tukey’s post-hoc test or Student’s *t*-test with *p* < 0.05 considered statistically significant.
